# An unusual presentation of a patient with severe hypogammaglobulinemia

**DOI:** 10.1002/ccr3.1877

**Published:** 2018-10-26

**Authors:** Thijs W. Hoffman, Diana A. van Kessel, Maarten J. D. van Tol, Gestur Vidarsson, Els C. Jol‐van der Zijde, Ger T. Rijkers, Heleen van Velzen‐Blad

**Affiliations:** ^1^ Department of Pulmonology St. Antonius Hospital Nieuwegein The Netherlands; ^2^ Division of Heart and Lungs University Medical Center Utrecht Utrecht The Netherlands; ^3^ Department of Pediatrics Leiden University Medical Center Leiden The Netherlands; ^4^ Department of Experimental Immunohematology, Sanquin Research and Landsteiner Laboratory, Academic Medical Center University of Amsterdam Amsterdam The Netherlands; ^5^ Department of Medical Microbiology and Immunology St. Antonius Hospital Nieuwegein The Netherlands; ^6^ Department of Science University College Roosevelt Middelburg The Netherlands

**Keywords:** antibody replacement therapy, hypogammaglobulinemia, immunodeficiency, primary antibody deficiency, vaccination response

## Abstract

We present a patient who was diagnosed with severe hypogammaglobulinemia after her newborn child presented with two episodes of meningitis. The patient had no history or symptoms suggestive of immunodeficiency. Thus far, a cause for the immunodeficiency has not been found, even after extensive immunological evaluation.

## 1 INTRODUCTION

Primary antibody deficiencies (PADs) are conditions characterized by hypo‐ or agammaglobulinemia and/or an impaired antibody response upon vaccination. The various types of PADs can range in severity from the virtual absence of immunoglobulins at a young age to adult‐onset low immunoglobulin levels to normal immunoglobulin levels with an impaired specific antibody response after vaccination.^1^ Patients with PADs most often present with recurrent or severe infections, or infections with unusual pathogens. Infections are commonly of the respiratory tract. The diagnosis of a PAD can be made after taking an infection history, performing immunological laboratory investigations, including measurement of the response to vaccinations, and excluding other causes of the observed immunological defects.^2^ Treatment for hypogammaglobulinemia most often is immunoglobulin replacement therapy with Intravenous Immunoglobulin (IVIG), prepared from pooled plasma of healthy adults. IVIG contains mostly immunoglobulin (Ig)G, and replacement therapy can lower infection frequency. 3

Here, we present an adult female patient with normal IgM and IgA serum levels who was coincidentally found to have a severe IgG deficiency because of serious infections in her newborn child. The patient was followed for more than sixteen years, and over the years various diagnostic investigations have been performed. The patient provided written informed consent for the case to be published.

## 2 CASE PRESENTATION

A child was born after a pregnancy of 38 weeks. The mother suffered from gestational diabetes during the pregnancy, which was otherwise uncomplicated. The child was male, had normal length and weight for his gestational age and had a good start. However, within two months, he suffered from two episodes of bacterial meningitis. Retrospective analysis of his blood, taken at three weeks of age (during the first meningitis episode), showed a virtual absence of IgG. As infants in the first months of life depend on the active transfer of maternal IgG across the placenta by the neonatal Fc‐receptor (FcRn),^4^ the mother was evaluated.

### 2.1 Initial history and examination

The mother reported a relatively uncomplicated medical history. She had been diagnosed with mild psoriasis when she was 14 years of age and had undergone an appendectomy at the age of 15. The psoriasis has been in remission since early adulthood. She did not smoke, had no known allergies, used no medications and did not have any complaints at the time of evaluation. With regard to infections, she reported one episode of severe cystitis requiring hospitalization at 10 years of age and one episode of pyelonephritis at 28 years of age, as well as having one episode of tonsillitis per year since more than ten years. She worked as an elementary school teacher.

Physical examination showed a healthy female, and no abnormalities were found. Specifically, there was no lymphadenopathy or splenomegaly and her palatine tonsils were relatively large but otherwise unremarkable.

### 2.2 Additional investigations

Laboratory analysis revealed severe hypogammaglobulinemia. Serum IgA level was 3.8 g/L, IgM was 0.4 g/L and IgG was 0.57 g/L. All IgG subclass levels were low. Serum albumin was normal. The results of immunological and additional laboratory investigations are shown in Table 1. Of note, there were no or very low circulating antibodies against specific antigens including diphtheria‐tetanus‐polio, despite a normal vaccination history. No other laboratory or immunological abnormalities were found. An HRCT‐scan of the thorax showed no abnormalities.

**Table 1:** Laboratory results at the time of diagnosis

Test	Result	Reference range
Erythrocytes (×10^12^/L)	4.4	3.8 ‐ 4.9
Ht (%)	41	36 ‐ 44
Hb (mmol/l)	8.6	7.7 ‐ 9.6
Leukocytes (×10^9^/L)	4.8	3.0 ‐ 10.0
Lymphocytes (%, absolute counts/μL)	35, 1700	20 ‐ 35, 1000 ‐ 2800
Thrombocytes (x10^9^/L)	152	150 ‐ 300
Serum protein level (g/L)	65	67 ‐ 81
Albumin (g/L)	40.4	36 ‐ 45
Serum protein electrophoresis	Normal	Normal

Blood type	A positive	
Anti‐B isohemagglutinin titer	1:16	≥1:8

Immunoglobulins		
IgM (g/L)	0.4	0.4‐2.5
IgG (g/L)	0.57	7.0‐17.0
IgG1 (g/L)	0.5	4.9‐11.4
IgG2 (g/L)	0.1	1.5‐6.4
IgG3 (g/L)	<0.1	0.2‐1.1
IgG4 (g/L)	<0.1	0.1‐1.4
IgA (g/L)	3.8	0.5‐3.7
IgA1 (g/L)	2.9	0.6‐2.4
IgA2 (g/L)	0.37	0.1‐1.6
Salivary IgA (mg/L)	>100	>60

Specific IgG antibodies		Sero‐positive cutoff
EBV VCA (titer)	<1:100	>1:100
CMV (AU/mL)	<0.5	≥15
Rubella (IU/mL)	<0.5	>10
Toxoplasma (IU/mL)	<0.5	>3

Autoimmune serology		Sero‐negative cutoff
ANA titer	<1:40	<1:40
RF	Negative	Negative

DTP antibodies		+3 wk	Protective levels
Diphtheria (Ig; AU/mL)	<0.01	1.42	>0.1
Tetanus (Ig; AU/mL)	0.02	>16	>0.1
Poliomyelitis type l (Ig; titer)	<1/2	≥1/4098	≥1:8
Poliomyelitis type II (Ig; titer)	<1/2	1/128	≥1:8
Poliomyelitis type III (Ig; titer)	<1/2	1/256	≥1:8

Pneumococcal antibodies		+3 wk	Protective levels
PPS3 (Ig; (U/mL)	6	107	≥20
PPS4 (Ig; U/mL)	3	19	≥20
PPS9 (Ig; U/mL)	1	20	≥20

Complement		Reference range
C3 (mg/dL)	133	90‐180
C4 (mg/dL)	30	15‐40
CH50 (%)	114	75‐125
AP50 (%)	129	78‐128

Lymphocyte subsets		
CD3+ (%, absolute counts/µL)	71, 1200	55‐83, 500‐2300
CD4+ (%)	42	28‐57
CD8+ (%)	29	10‐39
CD4/CD8 ratio	1.4	1.0‐3.5
NK‐cells (%, absolute counts/µL)	15, 250	7‐31, 90‐600
B‐cells (%, absolute counts/µL)	16, 275	3 −19, 100‐500

Reference ranges are based on the reference values used at the time of diagnosis. Absolute counts/µL were derived from leukocyte count and differential. EBV, Epstein‐Barr virus; CMV, cytomegalovirus; ANA, antinuclear antibody; RF, rheumatoid factor; DTP, diphtheria‐tetanus‐polio; PPS, pneumococcal polysaccharide Protective antibody level values are based on Plotkin^25^ for DTP‐vaccine, and on van Kessel et al^21^ for 3‐plex total anti‐pneumococcal antibody measurement. Vaccine responses (DTP, pneumococcal polysaccharide) were measured 3 wk after vaccination, before the patient received immunoglobulin replacement.

**Figure 1:**
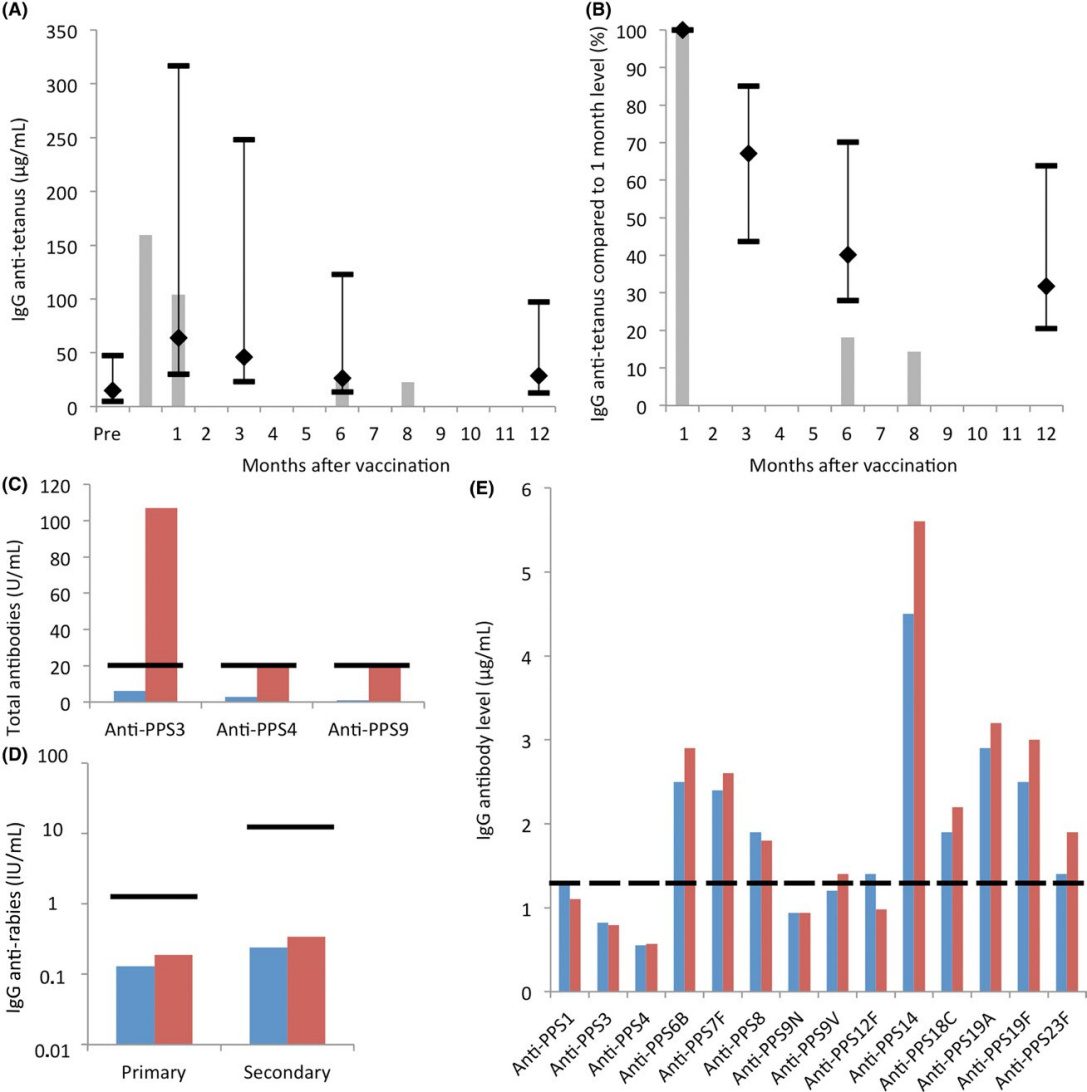
Antibody response to vaccinations. A, IgG anti‐tetanus antibodies before and after tetanus vaccination at the time of diagnosis. Gray bars represent antibody measurements in patient's serum samples. The black lines represent the IgG anti‐tetanus antibodies measured in 20 healthy adult donors (median, 5th, and 95th percentile). Samples were taken prior to the start of immunoglobulin replacement (samples at 3 and 5 wk post‐vaccination), and under immunoglobulin replacement (samples 6 and 8 mo post‐vaccination). B, IgG anti‐tetanus levels after tetanus vaccination, compared to levels 1 mo after vaccination. The patient's antibody level declined faster than in controls. The gray bars represent the patient, and the black lines represent healthy donors (median, 5th, and 95th percentiles). C, Response to 23‐valent pneumococcal polysaccharide vaccine at the time of diagnosis. The post‐vaccination sample was taken 3 wk after vaccination (red bars). Protective antibody levels are defined as ≥20 U/mL (horizontal lines). The vaccination response is deemed to be adequate in case of ≥2 out of three serotypes above this cutoff.^21^ D, Response to a purified chicken embryo cell rabies vaccine 6 y after diagnosis. The patient was receiving IVIG at this time, which did not contain anti‐rabies antibodies, as also evidenced by low pre‐vaccination antibody levels (taken just prior to a scheduled gift of IVIG). The patient received two vaccinations 3 mo apart, and antibody levels were measured before (blue bars) and 4 wk after (red bars) both vaccinations (just prior to a scheduled gift of IVIG). Normal values for vaccination response were based on median concentrations of anti‐rabies antibodies after vaccination of healthy adults.^22^ The normal median value was 1.9 IU/mL after the first and 18.1 IU/mL after the second vaccination (horizontal lines). E, Response to 23‐valent pneumococcal polysaccharide vaccine 11 y after diagnosis. The pre‐vaccination sample was taken just prior to a scheduled gift of IVIG (blue bars), the post‐vaccination sample was taken 3 wk later (1 wk prior to the next scheduled gift of IVIG, red bars). A normal response is defined as a two‐fold increase over baseline and values ≥1.3 µg/mL for at least 70% of serotypes (ie, 10 out of 14 serotypes)^2^

After the initial investigations, the patient was vaccinated with a 23‐valent unconjugated pneumococcal polysaccharide vaccine and diphtheria‐tetanus‐polio vaccine. Pre‐vaccination antibody levels were very low against all tested antigens (Table 1). Surprisingly, she mounted normal antibody responses against both polysaccharide and protein antigens. IgG anti‐tetanus antibodies showed a normal relative avidity (data not shown). Isotype analysis of anti‐pneumococcal polysaccharide antibodies showed relatively low levels of IgG antibodies five weeks after vaccination. Because of the very low circulating IgG levels, the patient was started on IVIG immediately after obtaining post‐vaccination blood samples (dosage 400 mg/kg/4 weeks). After the start of immunoglobulin replacement, IgG anti‐tetanus antibodies were still monitored for a period of 8 months. Compared to healthy controls, the patient showed an unusually fast decline of anti‐tetanus IgG antibody levels. After six months, the IgG antibody level was reduced to less than 20% of the original post‐vaccination antibody titer (Figure 1A,B).

### 2.3 Differential diagnosis and further investigations

At this point, we did not have an immunological diagnosis for our patient. The low IgG level and rapid decline of anti‐tetanus antibodies indicate an antibody deficiency. As there were no apparent other causes (eg, immunosuppressive medication, hematological malignancy, protein loss), our patient can be said to have a PAD. However, she did not fulfill the diagnostic criteria for common variable immunodeficiency (CVID),^5^ as she had normal levels of IgA and IgM. Furthermore, compared to other patients who have hypogammaglobulinemia but do not fit the criteria for CVID, our patient had extremely low IgG levels; these patients also usually have an impaired response to pneumococcal vaccine (after 3‐6 weeks), and not an initially normal response.^6^ Furthermore, almost all patients with PAD present with recurrent infections.^2^

**Figure 2:**
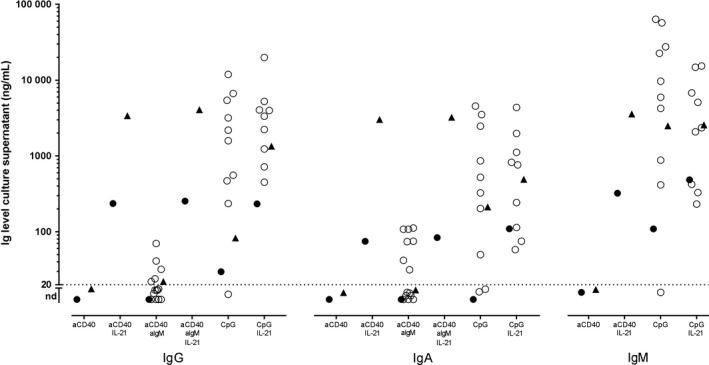
Immunoglobulin levels in culture supernatant after in vitro stimulation of PBMC acquired 16 years after diagnosis. PBMC were cultured for 7 d in the presence of the indicated (combination of) stimuli. IgG, IgA and IgM in the culture supernatant were quantified by sandwich ELISA. Stimuli: anti‐CD40: MAB89, 0.5 µg/mL; anti‐IgM: 1 µg/mL; CpG: ODN2006, 1 µg/mL, IL‐21:20 ng/mL. Triangles: patient (freshly isolated PBMC); filled circles day control (viably frozen PBMC); open circles: historic controls

Consequently, we considered causes of immunodeficiency that are less common, or as of yet, unknown. Therefore, we performed several additional investigations over the years. The first line of investigations was into defects in B‐cell or T‐cell development. Analysis of lymphocyte populations and B‐ and T‐cell differentiation stages was performed with flow cytometry on a blood sample obtained at 16 years after diagnosis (Table 2). The numbers of T‐, NK‐, and B‐cells and the distribution of T‐cell differentiation stages in the CD4+ and CD8+ T‐cell subsets were normal. Also, B‐cell distribution amongst the various differentiation stages was normal, including the numbers of circulating IgG isotype switched memory B‐cells and natural effector (marginal zone) B‐cells. In addition, after in vitro stimulation of peripheral blood mononuclear cells (PBMC) in the presence of IL‐21 and anti‐CD40+/− anti‐IgM or CpG, IgM, IgA, as well as IgG were produced (Figure 2).

**Table 2:** Lymphocyte and B‐cell subset analysis 16 y after diagnosis

	Percentage	Absolute counts/µL	Reference range (absolute counts/µL)
White blood cells (x10^9^/L)		5.4	4.0‐10.0
Lymphocytes		1200	1000‐2800
T‐lymphocytes		1110	700‐2100
NK‐cells		130	90‐600
B‐lymphocytes		230	100‐500
B‐cell differentiation stages	% of B‐cells		
Transitional B‐cells	6	13	3‐50
Naïve mature B‐cells	35.4	81	57‐447
Natural effector B‐cells	36.5	84	9‐88
Memory B‐cells	20.1	45	13‐122
IgM memory B‐cells	5.9	13	1‐33
IgG memory B‐cells	4.7	11	5‐59
IgA memory B‐cells	8.7	20	2‐35
Plasmablasts	0.2		
T‐cell differentiation stages	% of T‐cells/subset		
CD4+ T‐lymphocytes	59.7	700	300‐1400
Naïve	41.6		
Central memory	41.3		
Effector memory	16.3		
CD8+ T‐lymphocytes	38.1	400	200‐1200
Naïve	20.0		
Central Memory	13.1		
Effector Memory	66.3		
CD4/CD8 ratio		1.75	1.0‐3.5

Percentages of B‐cell differentiation stages are within CD19‐positive cells (=B‐cells). Percentages of CD4+ and CD8+ T‐lymphocytes are within CD3‐positive cells (=T‐cells). Percentages of CD4+ and CD8+ T‐cell differentiation stages are within the respective T‐cell subsets (CD4+ or CD8+). Markers used were as follows: CD3+ for T‐lymphocytes; CD16/56+ CD3‐ for NK‐cells; CD19+ for B‐lymphocytes; CD38 high CD24 high for transitional B‐cells; CD38 dim CD24 dim IgD+ CD27‐ for naïve mature B‐cells; CD38 dim IgD+CD27+ for Marginal zone/Natural effector B‐cells; CD38 dim IgD‐ CD27+ for memory B‐cells; plasmablasts CD45RO‐ CCR7+ CD27+ CD28+ for naïve T‐lymphocytes; CD45RO+ CCR7+ CD27+ CD28+ for central memory T‐lymphocytes; CD45RO+/− CCR7‐ for effector memory T‐lymphocytes.

**Figure 3:**
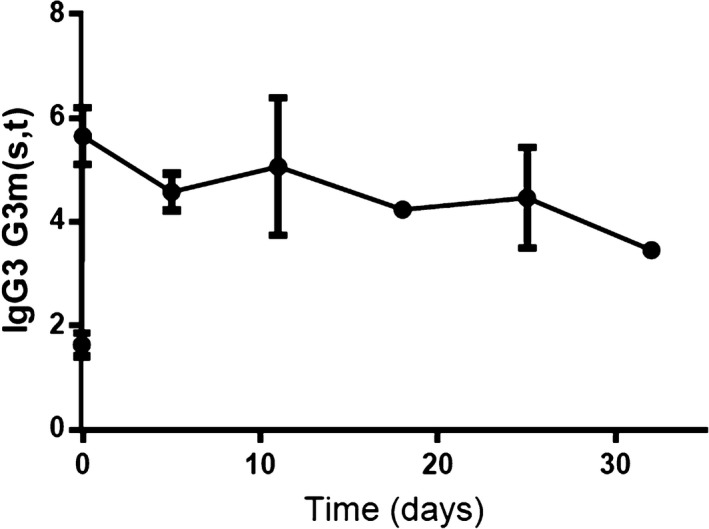
IgG turnover. Measurement of H435‐IgG3 levels after a gift of IVIG, 16 y after diagnosis. Data points represent means and standard deviations of multiple measurements (four dilutions in duplo per time point). The turnover of IgG from the gammaglobulin preparations was measured by the elimination of H435‐IgG3, a rare IgG3 allotype present in gammaglobulin preparations, but not endogenously produced by most people of Western‐European origin. People of Western‐European origin (including our patient) normally produce R435‐IgG3. R435‐IgG3 has a half‐life of one week due to diminished binding to FcRn, while H435‐IgG3 has an extended half‐life of three weeks like the other IgG subclasses, due to increased binding to FcRn.^23^, ^24^ The results were compatible with a normal IgG turnover, indicating a normal function of the FcRn in vivo

The second line of investigations was into IgG metabolism. FcRn is involved in IgG recycling and lengthens the half‐life of IgG subclasses 1, 2, and 4. FcRn does not bind IgG3, which therefore has a shorter half‐life (7 days vs 21 days).^4^ Patients with mutations in the β2‐microglobulin gene (whose protein associates with FcRn and is necessary for proper FcRn functioning) have been described, who presented with low IgG levels, a faster‐than‐usual decline in specific antibody levels after vaccination, and a relatively mild infectious history.^7^, ^8^ To test whether a defect in FcRn‐mediated functions could explain our patient's phenotype, we sequenced the *FcRn* gene as well as the *B2M* gene. No genetic defects were found in coding sequences. FcRn expression in monocytes was normal (data not shown). In addition, IgG turnover was found to be normal (Figure 3).

The third line of investigations was into the Fc‐gamma IIb receptor (FcγRIIb). This is one of the Fc‐gamma receptors, a class of receptors that bind to antibodies and regulate the immune response. FcγRIIb is the only inhibitory Fc‐gamma receptor and is the only Fc‐gamma receptor that is present on B‐cells. It controls the magnitude and persistence of antibody responses through effects on mature B‐cells, memory B‐cells, and plasma cells.^9^ In mice, over‐expression of FcγRIIb has been found to lead to reduced serum IgG levels and suppression of late IgG antibody responses.^10^ Therefore, we examined the expression of FcγRIIb on monocytes, as previously described.^11^ This was found to be normal. Furthermore, long‐range PCR of the *FcgammaR2b* gene^12^ was performed, which was also normal (data not shown).

Apart from these investigations, we have periodically monitored our patient for developing autoimmune disease or monoclonal gammopathy, with negative outcomes thus far. She also received additional vaccinations over the years. Six years after diagnosis, the patient was vaccinated with a rabies (purified chicken embryo cell) vaccine to investigate the response to a T‐cell dependent neo‐antigen. She showed an impaired antibody response to this vaccine upon primary vaccination as well as secondary vaccination 3.5 months later (Figure 1D). At the same time, she was booster vaccinated with the diphtheria‐tetanus vaccine, showing lower antibody responses than to the vaccination at the time of diagnosis. Eleven years after diagnosis, she was revaccinated with pneumococcal polysaccharide vaccine, as well as with diphtheria‐tetanus vaccine. She showed almost no change in antibody levels in response to both vaccines, as illustrated for pneumococcal polysaccharides (Figure 1E). In interpreting these responses, it should be kept in mind that the patient was receiving IVIG and therefore most if not all of the (pre‐vaccination) antibodies were passively administered. The impaired response after vaccination at 11 years after diagnosis, in contrast to the normal response at the time of diagnosis, was a reason to reconsider the diagnosis of a CVID. However, again our patient still cannot be diagnosed as having a CVID, since IgM and IgA levels are still normal.

Lastly, we investigated the patient's family members. There was no family history of autoimmune disorders, confirmed immunodeficiency, and recurrent or severe infections. Both of the patient's parents, her brother, and her three children are healthy. None of the family members report recurrent or severe infections. IgM, IgG, and IgA levels were normal in these family members (measured in the two oldest children directly after diagnosis of hypogammaglobulinemia in their mother and in all children and the parents and brother of the patient 16 years later).

### 2.4 Treatment

Our patient was started on immunoglobulin replacement with IVIG almost directly after she was found to have very low circulating IgG. At the time we did not want to risk our patient getting a severe or even life‐threatening infection due to her very low IgG level, despite having no recurrent infections before.

Four years after the start of immunoglobulin replacement, it was discontinued at the patients’ request. Discontinuation of IVIG led to a marked decline of IgG levels (from 7.1 to 3.2 g/L). Therefore, after two months, IVIG‐therapy was reinstated and IgG levels returned to the normal range. Until the present time, the patient has consistently received IVIG in a dosage of 400‐600 mg/kg/mo (at present 400 mg/kg/mo). During the follow‐up period, which currently spans more than 16 years, she has not reported serious or frequent infections. Presently, she is in excellent health. Although she did not have serious infections before IVIG, she has not had any episode of tonsillitis during IVIG, as opposed to yearly episodes before.

## 3 DISCUSSION

Here, we describe a 37‐year‐old female patient with a virtual absence of IgG in serum. This was a coincidental finding, because the patient was otherwise healthy and had no recurrent or unusual infections. There was no evidence for a defect in specific IgG synthesis or catabolism and no general protein loss. However, we did find a rapid decline of circulating specific IgG antibodies in response to tetanus vaccination. Further booster vaccinations given over the course of follow up for more than 16 years showed a diminishing response against tetanus and diphtheria. Additional investigations revealed that all differentiation stages of the B‐cell lineage were present in the blood in normal numbers and an intact functional IgG recycling system was demonstrated. IgM, IgG, and IgA were produced upon in vitro stimulation of B‐cells.

Apart from identifying the patient's condition as a PAD, we could not fit our patient within a specific diagnostic category. To the best of our knowledge, there are no comparable cases reported in the literature. As the hypogammaglobulinemia in our patient was a coincidental finding, and there was no clinical presentation suggestive of an immunodeficiency, there might be other apparently healthy humans with comparable immunological characteristics that have not yet been recognized.

It is unknown how long this patient had been hypogammaglobulinemic prior to the first immunological evaluation. She had yearly episodes of tonsillitis since more than 10 years prior to the start of IVIG, but no other infections. Her first and second child did not have severe infections in the neonatal period, so the hypogammaglobulinemia could have developed or worsened in between her second and third pregnancy. IgG levels are known to decrease during pregnancy and are lowest at term, albeit usually not below the normal range.^13^ In pregnant patients with CVID, the fetal IgG levels normally exceed the maternal levels.^14^ However, maternal IgG levels in these cases were higher than in our patient. In our case, maternal IgG levels might have been too low to be compensated for by active transport from mother to fetus.

One would expect that such a low IgG level in our patient would have led to more outspoken infectious manifestations. However, she did have a normal IgM and a high IgA (*ie*, high IgA1 and normal IgA2) serum level and initially showed an adequate antibody response after vaccinations. Therefore, it could be argued that she was relatively well protected against (mucosal) infections despite such a low circulating IgG level. It has been shown for CVID that normal numbers of class‐switched memory B‐cells are associated with less severe infectious manifestations and less organ complications.^15^ In contrast to most CVID patients, our patient had normal numbers of circulating germinal center‐derived memory B‐cells as well as marginal zone B‐cells.

Furthermore, NK cells and all differentiation stages in the CD4^+^ and CD8^+^ T‐cell subsets and in the B‐cells, including IgG^+switched^ memory B‐cells, were normal, suggesting a normal development of cells of the immune system. Also, B‐cell function in vitro was not impaired. This all points to a post‐germinal center defect in B‐cell development. A possible explanation for the presentation and laboratory findings in our patient might be a defect in the homing of IgG antibody‐secreting plasma cells to the bone marrow. In recent years, various factors that influence B‐cell differentiation to plasma cells, plasma cell homing to the bone marrow and plasma cell survival in the bone marrow have been discovered.^16^ Interestingly, in mouse models of defective plasma cell homing/survival, a vaccination response comparable to that of our patient is seen. In mice deficient in the known plasma cell survival factors Aiolos, CD93, or Zbtb20, the antibody response to vaccination is initially adequate, but rapidly declines.^17^, ^18^, ^19^, ^20^ As far as we know, the human phenotype of a plasma cell homing/survival defect has never been described. In order to find a potential cause for the defect in our patient, extensive genetic analyses could be performed in the future.

## 4 CONCLUSION

In conclusion, we present a patient that showed a profound hypogammaglobulinemia after her (third) newborn child presented with two episodes of meningitis and was found to have virtually no IgG in blood. The patient had no history of severe or recurrent infections. Immunological analysis revealed very low serum IgG levels, normal IgA and IgM levels and an initially adequate antibody response to vaccination with T‐cell dependent antigens that rapidly declined. No evidence for a defect in IgG catabolism was found. There were no abnormalities in T‐cell and B‐cell differentiation stages and production of IgG after in vitro stimulation of PBMC was normal. The patient has been doing very well under immunoglobulin replacement therapy for more than sixteen years.

## CONFLICT OF INTEREST

None declared.

## AUTHOR CONTRIBUTION

DAvK: was the patient's treating physician. TWH, DAvK, and HvVB: collected clinical data. MJDvT, GV, CMJvdZ, GTR, and HvVB: performed laboratory tests. All authors interpreted the outcomes. TWH and DAvK: wrote the first draft of the manuscript, and all authors revised the manuscript and agreed with the final form.
